# Moving Safely to Phase 2 of the COVID-19 Pandemic: What Is More Pressing, Dates or Data?

**DOI:** 10.5334/gh.813

**Published:** 2020-05-20

**Authors:** José Pablo Werba, Fabrizio Veglia

**Affiliations:** 1 Centro Cardiologico Monzino, IRCCS, Milan, IT

**Keywords:** COVID-19, cardiovascular, risk factors, algorithm, prevention, health policy, comorbidities

## Abstract

An in-depth analysis of gathered data collected in several countries on the pre-infectious characteristics of patients who have developed severe forms of COVID-19 disease could be the basis for developing tools to estimate individual risk and tailor protective measures for a safer route through the Phase 2 of the pandemic.

The rapid expansion of the COVID-19 pandemic has prompted health authorities and governments in different countries around the world to take measures that have progressively limited normal social life with the aim to reduce transmission, and thus the absolute number of serious cases and the risk of health systems failing to cope with the emergency. Initially uncertain of the infectiousness and lethality of COVID-19, these actions have been taken, as the authorities themselves have openly stated, ‘navigating by sight’, in the search for a so-called ‘proportionality’ between protecting citizens’ health and guaranteeing their individual freedom of study, work, movement and sociality [1].

The measures to contain the contagion have stiffened dramatically as the previous ones became openly insufficient. The virus has always run ahead of and faster than governments and citizens, uncertain of the real extent of the problem and reluctant to make draconian changes in their lifestyles. We entered the time of the pandemic in disorder and with great confusion. Everyone in the bunker was the ultimate directive, isolated from each other, and this is how we are forced to live today in order to stay alive, but above all, responsibly, in order not to transmit death. It seems to work, judging by the flattening of the curves of contagions, hospitalizations and mortality. However, like a broad-spectrum antibiotic, mass actions can have more relevant side effects than a more specific therapy. And every day this situation becomes more difficult to sustain, for individuals and for society as a whole.

Being uncertain about the real feasibility and timing for the development of effective therapies and vaccines against COVID-19 [2], the governments are working with virologists and epidemiologists to develop a Phase 2 of the battle against the pandemic, aimed at achieving a gradual resumption of socio-economic activity without jeopardizing the goals achieved so far. This is an unanimous expectation. However, in Italy, one of the European countries most strongly affected by the virus, as well as in the USA and other countries of the world, visions on the appropriate timing for reopening strongly diverge between government and opposition, between region and region, between state and state. A global health guide does not seem to be identified or recognized. Dates or Data? Some see the economic collapse as the most serious social risk, and demand definite dates for reopening. Others consider the health of the population as the priority asset, and demand certain data for a comprehensive evaluation of the situation that allows the implementation of suitable methods and procedures of protection.

However, in order to become useful, certain data emerged from the clinical experiences must be analysed critically and in depth, and the results of these analyses must be transferred into real instruments and actions. In this sense, one idea that experts and decision-makers could consider is the rationale and methodology long adopted for primary cardiovascular prevention, based on a combination of a general population and individual strategies.

At the general population level, the authorities are considering different initiatives, and several measures are likely to be implemented at the same time, including universal use of personal protective equipment, shift work, promotion of smart work, strengthening public transport, personal appointment services, guaranteed distance to customers in shops, contact tracing, virus detection and serological studies throughout the population, and so on.

At the individual level, instead, the development of an algorithm for the estimation of the risk of a serious event (need for intensive care, respiratory assistance or death), permanently updated according to new emerging data, could allow the identification of categories or clusters of patients with high vulnerability to which the most effective and rigorous individual protection measures (level of social distancing, characteristics of IPR, methods and frequency of monitoring) could be targeted. Analogously to the field of cardiovascular health [3], the algorithm would underpin the development of a tool (table or calculator) (Figure 1) that could be used by physicians and even by citizens themselves. Undoubtedly, to create and update such an algorithm would require numerous, complete and reliable data. And this is where a fundamental question arises: are these data available?

**Figure 1:**
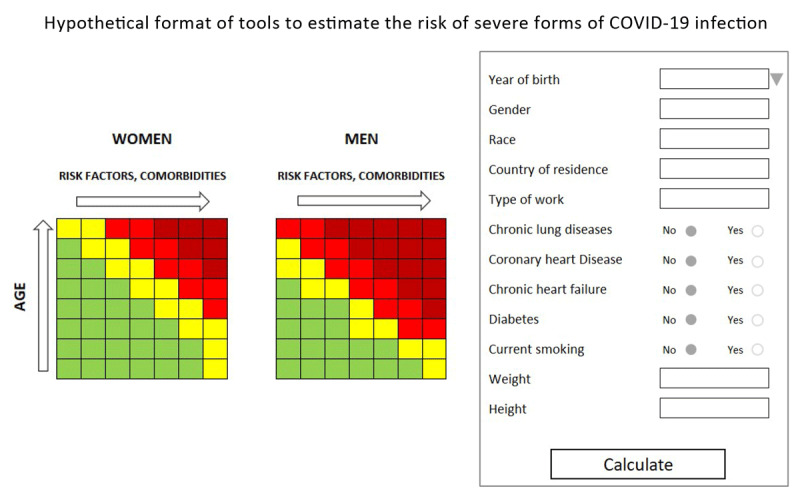
Further data are called-for to specify the suitable variables and the corresponding levels of risk.

Numerous studies published so far of patients hospitalized with COVID-19 infection [4,5,6,7] have revealed a long list of risk factors for severe disease and death, primarily the age of the patients and the presence of some comorbidities, including cardio and cerebrovascular disease and several cardiovascular risk factors such as diabetes, obesity and hypertension. However, as recently reported by Jordan RE et al. [8], these data emerge mainly from unadjusted analyses, which assess the associations between each condition and the risk of the outcome, one at a time. Since the prevalence and number of these comorbidities increase with age, assessing the independent weight of each of them is essential to know, for example, whether a young patient with diabetes or asthma shows a similar or higher risk than those of the same age without these conditions. Unfortunately, in most studies published so far that have carried out multivariate analysis, by the way in relatively small samples, some variables that are indicative of the severity of acute disease or of the extent of organ damage such as the occurrence of respiratory distress syndrome (ARDS), the SOFA (Sequential Organ Failure Assessment) score or the presence of high levels of troponin (hs-TNI) as an expression of cardiac damage have always been included in the adjustment models [9,10,11,12,13,14,15]. The inclusion of these variables, possibly of great value to stratify the risk of hospitalized patients, very likely results in an underestimation of the excess of risk associated with pre-infective characteristics and morbidity, which are the variables of interest in order to define a preventive policy for the community. Not surprisingly, in these multivariate analyses, many of the comorbidities associated in univariate analyses to COVID-19 related death (and even age in some cases) do not emerge as independent predictors of mortality. These results, only apparently contradictory, reflect a likely intermediate role of organ damage between the pre-infective characteristics and the unfavorable outcome. For example, in the study of Shi et al [13], in which hs-TNI at admission emerges as an independent predictor of death risk, the prevalence of pre-infective coronary heart disease in patients with high hs-TNI is almost five times higher than in patients without heart damage. All in all, it still remains unclear whether diabetes, hypertension and CVD are causally linked to severe COVID-19 infection or associated due to age [16].

Therefore, in order to build an algorithm that takes into account and properly ‘weights’ (Figure 2) the fundamental *pre-infectious characteristics* associated with the development of severe forms of disease and their possible interactions (age and comorbidity, but also perhaps ethnicity, latitude, urban or rural habitat, socio-economic condition, etc.), it would be necessary to bring together the raw data from different cohorts and make in-depth and homogeneous multivariate analyses in sufficiently large samples.

**Figure 2:**
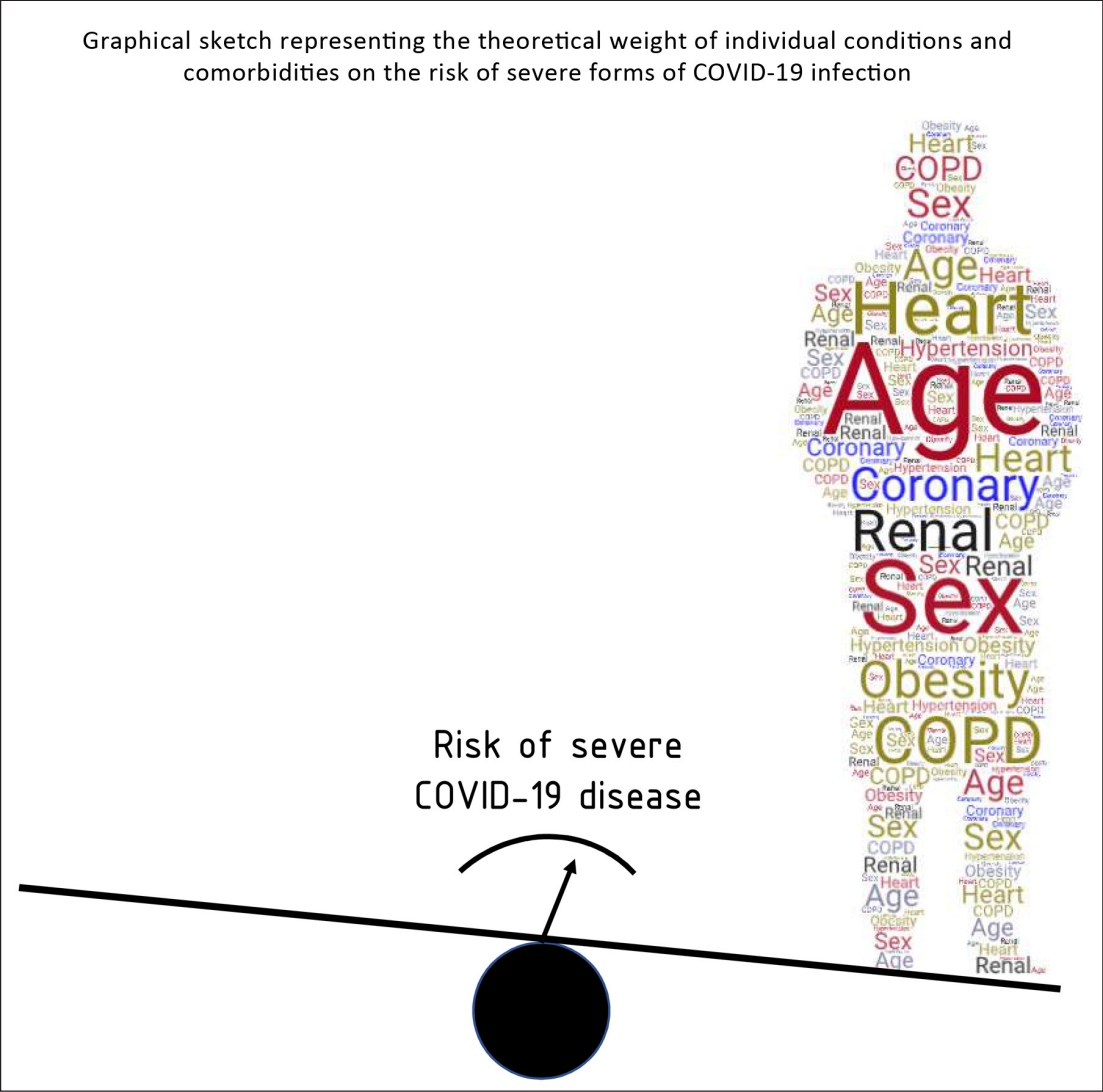
Size and repetitions of each subject’s feature represent their possible weight on the risk of the outcome. These weights are putative, as further data are needed to define the actual ones.

And here comes a second question: are the Global Health Agencies gathering and pooling these data across countries? Although in the World Health Organization’s document ‘A Coordinated Global Research Roadmap: March 2020’ [17], the activity ‘retrospective review of hospital admissions to identify risk factors for severe disease’ is declared a research priority, it is not listed among the ongoing global actions. It seems clear that in a pandemic situation the access of Global Health Agencies to demographic and clinical data of affected patients in different regions of the world should be guaranteed. Otherwise, the collection and analysis of data and the creation of tools for risk estimation will necessarily rely on national or local efforts only.

A few days ago, general recommendations for the management of severely ill adults with COVID-19 were published [18], as a first step towards the development of evidence-based guidelines for *the care of affected patients* [19]. It is desirable that the current information gaps in terms of individual and collective risk are filled as soon as possible and that this will enable the production of universal guidelines for the *prevention of COVID-19 infection* in the community, which may be crucial to restart the economy while saving lives [20].
